# Pancreatic β-cell heterogeneity in adult human islets and stem cell-derived islets

**DOI:** 10.1007/s00018-023-04815-7

**Published:** 2023-06-03

**Authors:** Noura Aldous, Abu Saleh Md Moin, Essam M. Abdelalim

**Affiliations:** 1 College of Health and Life Sciences, Hamad Bin Khalifa University (HBKU), Qatar Foundation, Education City, Doha, Qatar; 2 Diabetes Research Center, Qatar Biomedical Research Institute (QBRI), Hamad Bin Khalifa University (HBKU), Qatar Foundation, Education City, PO Box 34110, Doha, Qatar; 3 Research Department, Royal College of Surgeons in Ireland Bahrain, Adliya, Kingdom of Bahrain

**Keywords:** Diabetes, β-cell subpopulations, Cell–cell contact, Insulin secretion, Cell therapy

## Abstract

Recent studies reported that pancreatic β-cells are heterogeneous in terms of their transcriptional profiles and their abilities for insulin secretion. Sub-populations of pancreatic β-cells have been identified based on the functionality and expression of specific surface markers. Under diabetes condition, β-cell identity is altered leading to different β-cell sub-populations. Furthermore, cell–cell contact between β-cells and other endocrine cells within the islet play an important role in regulating insulin secretion. This highlights the significance of generating a cell product derived from stem cells containing β-cells along with other major islet cells for treating patients with diabetes, instead of transplanting a purified population of β-cells. Another key question is how close in terms of heterogeneity are the islet cells derived from stem cells? In this review, we summarize the heterogeneity in islet cells of the adult pancreas and those generated from stem cells. In addition, we highlight the significance of this heterogeneity in health and disease conditions and how this can be used to design a stem cell-derived product for diabetes cell therapy.

## Introduction

Pancreatic β-cells are glucose-regulated insulin-producing cells located in islets of Langerhans in the pancreas. These cells play an essential role in glucose metabolism and regulation. Reduction in β-cell function and/or mass can lead to alterations in glucose metabolism leading to abnormal elevation in blood glucose levels and eventually causing diabetes. Different therapeutic approaches for diabetes aim to maintain/restore the β-cell mass, hence function, including whole pancreas or pancreatic islet cell transplantation [1]. However, with islet transplantation being the ideal treatment for type 1 (T1D) and end-stages of type 2 diabetes (T2D), it remains difficult due to the limited number of donors and the need for the usage of strong immunosuppressant drugs [2]. Moreover, pancreatic islet cell transplantation requires at least 10,000 islet equivalents per kilogram of body weight which can be obtained from 2–3 donors approximately [3]. This also adds up to the limited resources for treating more patients with diabetes in need for such interventions. New research approaches aim to efficiently generate an unlimited source of functional pancreatic β-cells in vitro using human pluripotent stem cells (hPSCs) [4]. hPSCs are a valuable tool for generating different types of cells, including pancreatic islets and β-cells. As the structure of pancreatic islets is essential for proper cell-to-cell interaction and proper regulation of insulin secretion, multiple efforts focus on understanding the complex connective network between the different endocrine cells [5].

Recent studies have described that not all pancreatic β-cell populations are similar but heterogenous. To gain more insight, one should consider the factors that could contribute to β-cell heterogeneity. Hence, understanding the process of β-cell development, β-cell interaction with neighbouring cells, and β-cell response to stimulus can open the door to an explanation behind β-cell heterogeneity.

## Islet structure and composition

The adult endocrine pancreas, islets of Langerhans, constitutes about 1–4% of the total pancreas [6]. Each islet contains hormone-producing cells that synthesize and release glucagon (GCG; α-cells), insulin (INS; β-cells), somatostatin (SST; δ-cells), pancreatic polypeptide (PPY; γ-cells) and ghrelin (GHRL; ε-cells) in a nutrient-dependent fashion [7, 8]. Pancreatic islets receive up to 20% of the total pancreatic blood supply, hence, they are defined as highly vascularized micro-organs [9]. In addition, studies have shown that the microvascular density of pancreatic islets is 5–10 times higher than that of surrounding exocrine tissues [10]. The increased islet vascularization helps in sensing the systemic blood glucose levels, which is essential for the proper accomplishment of the islets’ endocrine function and efficient hormone delivery to target tissues [9, 11]. It has been reported that islets are not simply disordered clusters of endocrine cells but are highly organized micro-organs with a species-specific three-dimensional architecture. This unique cellular organization allows pancreatic islets to effectively carry out their primary physiological function of responding to changes in metabolic demands and regulating optimal glucose levels in the bloodstream. Furthermore, physical, and electrical cell–cell coupling enable synchronous hormone secretion, and intra-islet paracrine signaling is directed and connected with the nervous system for feedback regulation [12–14].

The architecture of mouse islets is characterized by a central, rounded core primarily composed of insulin-secreting β-cells. Meanwhile, the islet periphery is mainly occupied by α-cells that secrete glucagon, δ-cells that secrete somatostatin, and γ-cells that secrete polypeptide [15]. The β-cells located in the central core of the islet form rosette structures that are polarized around the blood capillaries [16]. Within these rosettes, β-cells have an apical domain located on the outer edge of the rosette, where primary cilia extend into the shared extracellular spaces. The basal domain of the β-cell is located adjacent to the blood vessel, where the insulin granules are located. The lateral domains between the edges of β-cells are enriched in glucose transporters and Ca2 + sensing machinery [16–18]. The architecture of human islets is characterized by a higher degree of complexity. There are several proposed models for the “stereotypical” human islet, each with its unique features [19–21]. The proportion of the primary endocrine cell types within the islet varies between mice and humans. In mice, approximately 75–80% of the islet cells are β-cells, with 20% α-cells, and less than 10% δ-cells. In contrast, the islets in humans have a lower proportion of β-cells, ranging from 55 to 70% and a higher percentage of α-cells, ranging from 30 to 45%, with less than 10% for both δ- and γ-cells [19, 20, 22–24]. The size of human islets can vary significantly, with a diameter range of approximately 50–500 μm and an average of 1500 cells per islet. Variations in the ratio of endocrine cells among individuals are reflected in differences in β-cell mass, [25, 26], while the distribution of islet cells across pancreatic regions exhibits only minor differences [27]. Differences in the methods used to study human islet architecture have resulted in various proposed models, and it is uncertain whether a stereotypical architecture exists in humans at all [11, 26]. However, research supports the existence of a common design principle between mice and humans, wherein homotypic interactions among the same type of endocrine cells are more crucial than heterotypic interactions between different types of endocrine cells [26, 28, 29]. Recent findings suggest that there are also conserved β-cells polarity domains in both species, but their functional roles in islets remain unclear and require further investigation [18, 30, 31].

## Heterogeneity of pancreatic β-cells

Cumulative evidence showed the existence of heterogenous β-cell sub-populations. By implementing new biomarkers and using advanced single-cell analyses, the identification of the different β-cell sub-populations became more promising. Initially, β-cell heterogeneity has been suggested through the identification of various response levels to glucose observed by different INS biosynthesis and secretion patterns [32]. Additional β-cell heterogeneity aspects include differences in maturity and proliferative states, redox states, membrane potentials, and glucose transport [33]. Different studies have revealed β-cell heterogeneity in animal and human models. However, here, we only focus on the identified β-cell populations in humans.

## Maturation and proliferative heterogeneity

Proper glucose response and INS secretion require mature β-cells that can synthesize sufficient amounts of INS upon glucose uptake. Immature β-cells are marked with higher proliferation and metabolic demands for the energy-consuming processes during cell proliferation [34]. Unlike immature β-cells, mature cells become more function-specific and less proliferative. Interestingly, the existence of both mature and immature β-cell populations has been discovered using single-cell analysis techniques. Wang et al. revealed three β-cell clusters (C1, C2, and C3) with different proliferative capabilities [34] (Table 1). C1 showed a quiescent cell state with no proliferation, while C2 and C3 revealed higher proliferative rates indicated by the expression of proliferation marker Ki67^+^ [34]. Interestingly, C1 showed high PDX1 and INS expression and is found to be increased with age but decreased with obesity. Proliferative C2 and C3 express a set of genes important for cell adhesion and migration including CD44, CD9, CD49F, and CYP26A1. Furthermore, C2 showed higher phosphorylation levels of PDGFRA, pERK1/2, pSTAT3, and pSTAT5. Regression models revealed that C2 negatively correlates with T2D, while C3 negatively correlates with age [34]. Several other studies support the presence of different β-cell populations depending on their maturity level. Szabat et al. identified two β-cell populations with distinct gene profiles: PDX1^+^/INS^low^ and PDX1^high^/INS^high^ [35]. PDX1^+^/INS^low^ cell population exhibits immature β-cell characteristics, associated with upregulated developmental, proliferative, and apoptotic markers (Table 1). Since PDX1^+^/INS^low^ are immature cells, β-cell function associated genes are remarkably reduced and cells reflected progenitor-like phenotype with polyhormonal gene profile by expressing PPY, GCG, SST, and GHRL. Remarkably, the expression of calcium-modulated INS secretion regulators (CAMK2G and MAPK4) is increased. However, MAPK1, a regulator of INS secretion in response to glucose, is decreased in PDX1^+^/INS^low^ cell population [35]. Furthermore, a few diabetes-related genes are enriched in PDX1^+^/INS^low^ cells as well including DPP4, LEPR, and SIRT5. Syntaphilin (SNPH), an inhibitor of SNARE complex formation and exocytosis regulator, is also increased [35]. PDX1^high^/INS^high^ population composed the majority of the β-cell population (78.3%) and showed mature β-cell characteristics with increased INS secretion. This cell population has high levels of mature β-cell function-related genes including GLUT2, MAPK1, CALML4, TIPRL, G6PC2, IAPP, and GCGR. Yet, SIRT1, a diabetes-related gene that has been shown to enhance INS resistance and diabetes, is found to be higher in PDX1^high^/INS^high^ [35, 36]. Even though PDX1^high^/INS^high^ are mature cells, development-related genes in TGFβ superfamily are also enriched such as BMP5, GREM1, and TGFBR3. Conversely, PDX1^+^/INS^low^ cells have increased expression of FSTL5 and BAMBI which are from the same TGFβ superfamily [35]. Nevertheless, mature cells display enriched maturity-related genes like GCGR, IAPP, and MAPK1 [35].

**Table 1:** Beta cell subpopulations identified in human pancreatic islets

Beta cell subpopulation (INS +)	Gene expression profile	Beta cell maturity	Beta cell functionality	Percentage	In diseased and normal conditions	Used method	References
ST8SIA1^−^	High GLUT2 expression	Mature	Improved insulin secretion	~ 85%	Reduced in T2D	FACS live sorting (HPi2 + /HPa3-)	Dorrell et al. [37]
ST8SIA1^+^	Low GLUT2 expression High K + channel expression High f-channel HCN1 SIX3, RFX6, MAFB and NEUROD1	Immature	Reduced insulin secretion	~ 15%	Increased in T2D		
PDX1^high^/INS^high^	GLUT1, MAFA, MAFB, BMP5, TGFBR3, GREM1, DACH2, IRX3, DLK1, FOXN2, ITGB7, GCGR, IAPP, CAPN13, G6PC2, TIPRL, CALML4, MAPK1, SIRT1, REG1A, BMF, CFLAR, DDX58, SLC2A2, SLC6A6, SLC6A15, SLC17A6, SLC39A11, SLC38A6, SLC16A10, SLC25A19	Mature	Improved insulin secretion	78.3%	–	*Pdx1*mRFP-*Ins1*eGFP dual reporter lentiviral vector	Szabat et al. [35]
PDX1^+^/INS^low^	PRG-3, IRX2, MSI2, VIL1, RFX2, BAMBI, FSTL5, VIM, FOXP4, CD39, MUC13, LAMB1, SNPH, CAMK2G, MAPK4, PPY, SST, GHRL, SORBS1, DPP4, LEPR, SIRT5, TRIB3, CHN2, FAP, TM4SF4, PRP4A3, ALK, CARD11, DAP, DFFA, SLC5A1, SLC40A1, SLC44A3, SLC22A4, SLC7A14, SLC35F4	Immature, Increased proliferation, a small percentage of these cells slowly mature into PDX1^high^/INS^high^ (9.3%)	Reduced insulin secretion	21%	–		
INS^lo^ UPR^lo^	ID1, ID3, ID4, JUNB, CEBPD, SIX3, TSHZ2, FOS, EGR1, MLXIPL, NFIC, YBX1, KLF6, ZFP36L2, MLLT11, SIX3, HES1, KMT2E, TSC22D1, ISL1, NKX2-2, GTF3A, ZNF580, NEUROD1	–	Low insulin secretion	16%	Increase after stress resolve	Single-cell RNA sequencing and pseudotime analysis	Xin et al. [41]
INS^hi^ UPR^lo^	SOD3, MAFA	–	High insulin secretion	38%	Decrease in stress conditions and insulin demand		
INS^lo^ UPR^hi^	SOD1, SOD2, CD44, GCLM, GSTP1, GLRX2, GLRX3, GPX1, GPX2, GPX3, GPX4, SLC3A2, PRDX1, PRDX2, PRDX6, TXN, TXNL1, TXNRD1, NQO1, FTH1, FTL, PCBP1, SQSTM1, PARK7, CDKN1A, MANF, ID1-3, ID2, ATF3, ATF4, CREM, JUN, NFKBIA, TBPL1, ELF3, DBP, ZNF622, ARID5B, ENO1, HMGA1, NR0B1, CEBPG, DDIT3, NR3C1, TSC22D1, ZNF511, TAX1BP1, CDC5L, LYAR, PHF5A, ZC3H15, ZBTB2, DNAJC2, ZNF410, TFB2M, ZNF830, ZNF22, MAF1, ZNF593, GTF3A, DRAP1, PHB, SFPQ, YBX1, KLF6, HES1, BHLHE40, ZNF331, ZNF326, CAMTA2, ZNF143, EGR4, YBX3, MATR4, CREB3, CEBPB, HES4, ZNF207, SMARCE1, XBP1, ZNF706	Increased proliferation and metabolic demands	Low insulin secretion	~ 23%	Increase in stress conditions and high insulin demands		
C1	Higher PDX1 expression*	Quiescent	High insulin expression*	10–70%	Increase with age and decrease with obesity	Mass cytometry	Wang et al. [34]
C2	CD44 ^high^, CD49F ^high^, CD9, CYP26A1, PDGFRA, pERK1/2, pSTAT3, pSTAT5, Ki67	Increased proliferation	–	5–55%	Decrease in T2D		
C3	CD44, CD49F, CD9, CYP26A1, Ki67	Increased proliferation	–	20–100%	Decrease with age		
C1	RBP4, FFAR4, ID1, ID3	–	Correlate with insulin resistance	~ 22%	Increase in obese and T2D individuals	single-cell RNA-sequencing	Segerstolpe et al. [43]
C2	ID1, ID3	–	–	~ 7%	–		
C3	ID1, ID3	–	–	~ 19%	–		
C4	ID2	–	–	~ 13%	–		
C5	RBP4, FFAR4	–	Correlate with insulin resistance	~ 39%	Increase in obese and T2D individuals		
β_HI_	High nuclear compaction High H3K27me3 expression High insulin content High CD24 surface marker expression High mitochondrial content High insulin secretory function	Increased proliferation	–	~ 30–90%	Increase in T2D	FACS, single-cell RNA-sequencing, and SCAN-seq	Dror et al. [54]
β_LO_	Low H3K27me3 expression Low CD24 surface marker expression	–	–	~ 10–70%	–		
CD24^high^	Highest CD24 surface marker expression High INS and SST expression	–	–	~ 1%	–		

*UPR* unfolded protein response, *C* clusters identified

Other research groups have identified β-cell heterogeneity according to surface markers expressed on β-cells. Dorrell and colleagues have categorized β-cells into four distinct populations based on two surface markers (CD9 and ST8SIA1) [37]. Interestingly, ST8SIA1^−^ cells, composing ~ 85% in healthy individuals, express high GLUT2 levels and show more mature phenotypes regarding β-cell functionality. By contrast, ST8SIA1^+^ cells show immature cell phenotypes and compose ~ 15% in healthy individuals. Additionally, immature cells had lower INS secretion profile and decreased GLUT2 expression [37]. Yet, ST8SIA1^+^ cells have increased K^+^ channel expression and a three-fold increase in f-channel HCN1 expression, a possible gene with INS secretion relevance [37]. Several transcriptional factors (TFs) are expressed in ST8SIA1^+^ as well, such as SIX3, RFX6, MAFB, and NEUROD1. SIX3 has been identified as an important TF in age-related β-cell maturation and expression levels positively correlate with INS secretion [37, 38]. Moreover, the ST8SIA1^−^ cell population is reduced in T2D, while the ST8SIA1^+^ cell population increases in the case of diabetes [37]. Recently, CD9 has been identified as a negative surface marker for functional glucose-responsive β-cells. hPSC-derived pancreatic β-cells, which are negative for CD9, have higher NKX6.1, MAFA, and C-peptide levels in comparison to CD9^+^ cells [39].

The hallmark of a mature β-cell involves a complex cellular identity with finely tuned coupling to the prevailing glucose level. Ultra-structurally, a β-cell is estimated to contain ~ 10,000 dense-core secretory granules with a clear peripheral mantle and the presence of fully processed proinsulin molecules in these dense-core granules depends on the maturity of the β-cell [40]. The concerted activity of key TFs determines the fate of endocrine cells; hence, mature β-cells are also distinct in terms of the expression of certain genes and TFs. Single-cell analyses that have revealed the transcriptional program of human pancreatic endocrine cells depicted genes such as PAX4, PDX1, MAFA, MAFB, DLK1, SIX2/3, ID1, IAPP, UCN3, and OLIG1 that are highly or exclusively expressed in human β-cells [41, 42]. Interestingly, those studies did not report any sub populations of islet cells. In addition, single-cell transcription profiling in human islets also revealed two TFs, SIX2 and SIX3 [43–45], which have been shown to enhance INS content and secretion in immature β-cells, suggesting their crucial role in human β-cell maturation [38].

## β-cell heterogeneity under stress and diabetes conditions

Previous studies have identified relationships between β-cell populations and diabetes. The heterogeneity of β-cells has been found to be changed under diabetic conditions where studies have reported changes in β-cell sub-populations in pancreatic islets derived from T2D patients. This alteration suggested a transformation of β-cells into progenitor cells or other endocrine islet cells (reviewed in [46]). Wang et al. have identified three β-cell clusters (C1, C2 and C3) where C2 and C3 reflect immature phenotypes and C1 reflects mature phenotypes. However, the C2 cell population decreases in the presence of T2D [34]. Unlike C2 immature cells, the immature ST8SIA1^+^ cell population increases with T2D [37]. Another study conducted by Segerstolpe et al. identified five β-cell clusters (C1, C2, C3, C4, and C5) [43]. Notably, C1 and C5 characterized by the expression of RBP4 and FFAR4, which are diabetes-associated genes and correlate with INS resistance, are found to be increased in T2D and obese individuals [43, 47, 48]. Few studies have identified β-cell heterogeneity in diabetes conditions, yet more single cell studies performed on human islets in both healthy and diseased conditions are required.

Synthesized proteins undergo protein maturation and folding in the endoplasmic reticulum (ER). The amount of protein synthesis is regulated by the folding capacity within the cell’s ER. In normal β-cell conditions, > 10% of protein production accounts for INS synthesis and this percent increases to 50% in stimulated conditions. Nevertheless, proinsulin is widely known as a misfolding-prone protein where 20% of synthesized proinsulin fails to fold properly [49]. In cases of increased INS demands as in diabetes, accumulation of misfolded proinsulin protein can result in ER stress in β-cells [50]. Unfolded protein response (UPR) is a homeostatic cell signaling-based system that recovers ER function and folding capacity; however, the inability to adapt to ER stress causes cell apoptosis [51]. Another factor to β-cell stress is the accumulation of reactive oxygen species (ROS) known as oxidative stress. β-cells depend mainly on oxidative phosphorylation for adenosine triphosphate (ATP) synthesis, and this add on the stress levels exerted on β-cells. Furthermore, β-cells possess low defence mechanisms against ROS that further increase their vulnerability to stress condition formation [49]. However, a recent study has revealed a unique and dynamic mechanism of β-cell heterogeneity where it allows the cells to defend themselves and adapt to various insults. With the aid of single-cell and pseudo-time analyses, three β-cell populations have been identified in stress conditions: INS^lo^ UPR^lo^, INS^hi^ UPR^lo^, and INS^lo^ UPR^hi^ [49]. A sequential order of these cell populations is suggested where a proportion of β-cells characterized by high INS synthesis and low UPR (INS^hi^ UPR^lo^), supports the body’s need for INS. Meanwhile, another cell proportion characterized by low INS synthesis and high UPR (INS^lo^ UPR^hi^) would be allowed to complete its stress recovery. INS^lo^ UPR^hi^ has high expression of proliferation-related genes and they have high metabolic demands observed by the increased expression of G6PD, RPIA, TALDO, and TKT. An intermediate cell population marked as INS^lo^ UPR^lo^ can be observed after stress recovery and before initiating the increased INS synthesis demands [49]. Whether these β-cell states under stress conditions are permanently present throughout life or have a limited capacity for activating UPR are still unknown. Furthermore, Muraro et al. have identified a distinct β-cell population marked by the increased expression of SRXN1, SQSTM1, FTH1P3, FTH1, and FTL in which they labelled them as FTH1^+^ cells [44]. These highly expressed genes are associated with ER and oxidative stress responses [52, 53]. This further supports the concept of β-cell heterogeneity under stress conditions.

## Epigenetic heterogeneity of β-cells

Interestingly, β-cell heterogeneity has been identified at epigenetic levels that affect β-cells’ transcriptomic profiles. A recent study conducted by Dror et al., has identified two β-cell subtypes with high (β_HI_) and low (β_LO_) expressions of H3K27me3, a polycomb-associated heterochromatin, where β_HI_ have an average of ~ 4.5 folds increase in H3K27me3 expression level [54]. Furthermore, the β_HI_ cell population shows enriched active nuclear interior and smaller nuclei compared to β_LO_ cells, which is consistent with H3K27me3 role in chromatin silencing and compaction [54, 55]. Transcriptomic analysis revealed increased expression of CD24 surface marker in β_HI_ population. INS + β-cells co-sorting using H3K27me3 and CD24 revealed two distinct populations as well where ~ 20% of all INS + cells were CD24 + and ~ 80% were CD24- [54]. A rare subtype (~ 1%) of extremely high CD24-expressing cells (CD24^high^) was also identified with high SST expression, supporting previous studies reporting high CD24 expression in δ-cells [54, 56]. In addition, β_HI_ cells have higher mitochondrial content, increased INS secretory function, and a small but significant increase in proliferative capacity. Differential DNA methylation analysis between β_HI_ and β_LO_ revealed enrichment in specific enhancer regions and H3K27me3 annotated genomic regions; at regions binding JUNB, AEBP2, CEBPD, MAFB, ATF3, H3K3me1, and PROX1 [54]. Rs340874 is a significant T2D-associated variant located in the PROX1 locus in human, suggesting possible cell-subtype-specific effects due to the variant [54]. In contrast, β_LO_ cells exhibit hypermethylation in NKX6.1 and NEUROD1 regions, potentially indicating a more thorough decommissioning of the plasticity linked to NKX6.1 and NEUROD1 in β_LO_ cells [54]. Interestingly, single-cell RNA-seq analysis in the context of T2D revealed 2 major cell clusters distributed along β_HI_ and β_LO_ axis based on their differentially expressed genes [54]. Identified β-cell populations from T2D patients showed increased β_HI_/β_LO_ ratio, proposing a diabetes-specific alteration in β_HI_/β_LO_ ratio.

## Cell–cell communication and its importance for β-cell functionality: knowledge from both rodent and human β-cell studies

**Fig. 1:**
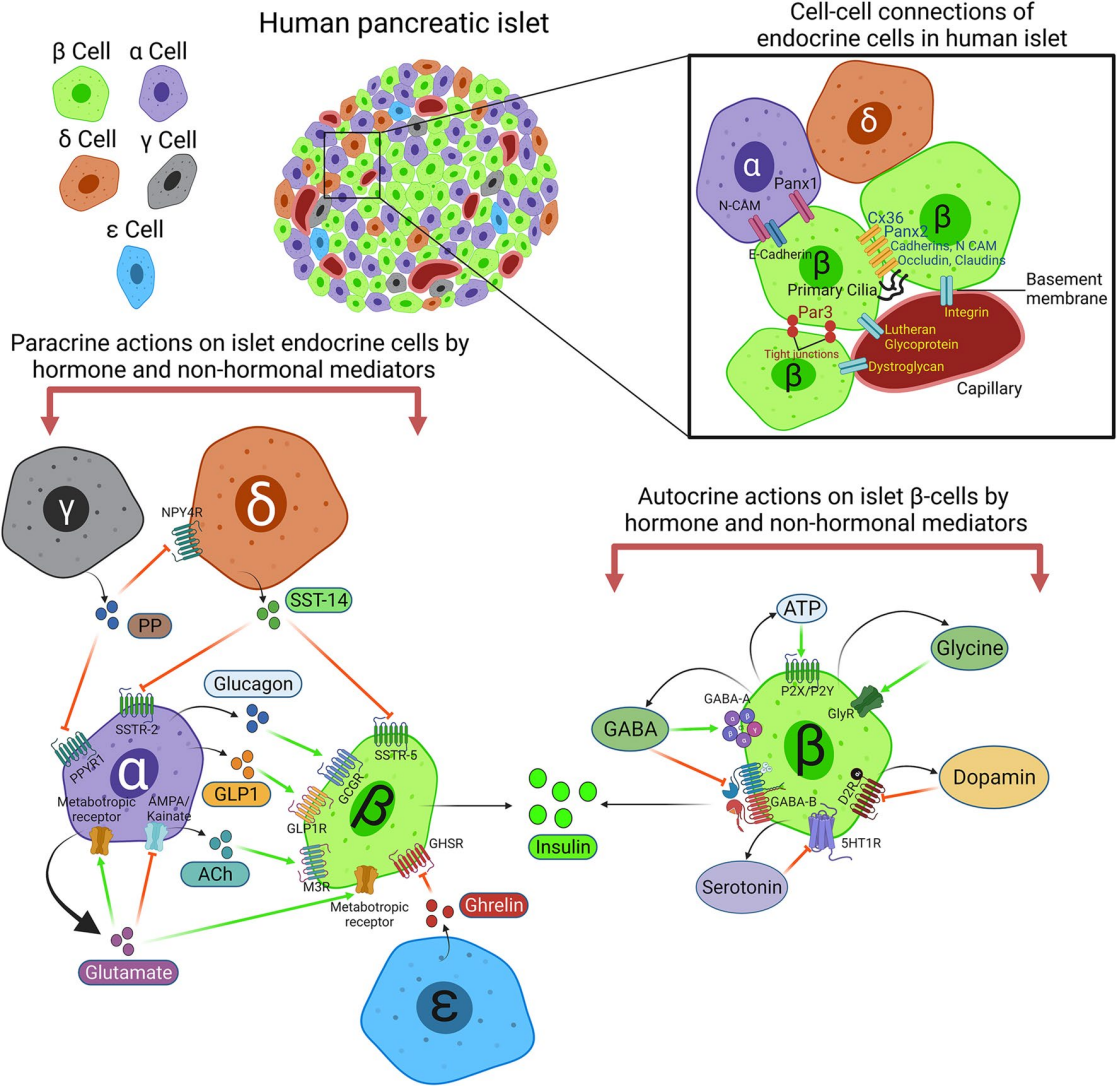
Organization and interactions of endocrine cells in human pancreatic islets and the autocrine and paracrine modulators of endocrine cells’ function: Top left panel, Cellular compositions of human islets containing insulin-producing β-cells (green cells), glucagon producing α-cells (purple cells), somatostatin producing δ-cells (orange cells), pancreatic polypeptide producing PP(γ) cells (grey cells) and ghrelin producing ϵ-cells (blue cells). Top right panel, Cell–cell connections of islet endocrine cells (inside the black square). Bottom left panel, Paracrine actions of islet endocrine cells by hormone or non-hormone mediators. Hormones and non-hormone biomolecules are released from one endocrine cells, bind to the specific receptors expressed on the cell surface of neighbouring endocrine cells to exert paracrine actions. Bottom right panel, the illustration depicts the autocrine actions of hormones and non-hormone medicators on β-cells. Hormones and non-hormones biomolecules that are secreted from the β-cells, bind to the respective receptors of the same cells to exert their autocrine function. *N-CAM* neural cell adhesion molecule, *Panx1* Pannexin1, *Panx2* Pannexin2, *Cx36* Connexin 36, *Par3* Par family protein 3, *NPY4R* Neuropeptide Y receptor subtype 4, *PPYR1* pancreatic polypeptide receptor subtype 1, *SST-14* Somatostatin-14, *SSTR* somatostatin receptor,* AMPA* α-Amino-3-hydroxy-5-methyl-4-isoxazolepropionic acid, *GLP1* glucagon like peptide-1, *Ach* acetylcholine, *GLP1R* GLP-1 receptor, *GCGR* Glucagon receptor, *M3R* muscarinic receptor, *GHSR* growth hormone secretagogue receptor, *ATP* adenosine tri-phosphate, *GABA* gamma amino butyric acid, *P2X/P2Y* purinergic receptor 2X/2Y, *GABA-A/B* gamma amino butyric acid receptor A/B, *GlyR* glycine receptor, *5HT1R* 5-hydroxy tryptamine receptor 1, *D2R* dopamine receptor 2

The optimum function of β-cells in pancreatic islet requires a tight and temporal control of INS secretion, which is largely achieved by sensing and integrating a variety of signals including circulating glucose, hormones, neurotransmitters, and other nutrients [57, 58]. However, local regulation may also take place within islets, in which β-cells cohabit with several other cell types and communicate with neighbouring cells (plausibly over long distances) to organize their activities. As discussed in the previous section, in human islets, β-cells are interspersed with other endocrine cells (largely α-cells) and promote a heterologous contact of endocrine cells [19]. Such divergence in islet architecture influences cell–cell communication among the islet cells (summarized in Fig. 1).

## Cell adhesion molecules

### Gap junctions

The best-characterized cell–cell coupling mechanism in the pancreas is provided by gap junctions (GJs). GJs are clusters of intercellular channels that allow the direct transfer of ions fluxes and second messengers between adjacent cells. In vertebrates, these channels are mainly formed by a family of proteins called connexins (Cxs), composed of 20 members in mice and 21 in humans [59]. Most β-cells in rodent and human islets have been found to be electrically coupled by connexin 36 (Cx36 or GJD2) facilitating the rhythmic and synchronized bursts of electrical activity and Ca^2+^, which are observed under both resting conditions and following stimulation of INS secretion by physiological nutrients and drugs [60, 61]. Cx36 is critical for coordinating islet activity as dispersed β-cells fail to synchronize their responses to glucose, thus restricting β-cells’ proper function, particularly the pulsatility of [Ca(2 +)](i) and INS secretion during glucose stimulation [62]. Even though other islet cells (for example, α- and δ-cells) show oscillations (of GCG and SST respectively) [63, 64], it has been suggested that they are also coupled. However, the coupling between other islet cells does not involve CX36 as it forms only homomeric and homotypic intercellular channels (specific only to β-cells) [65, 66]. Another junction protein, pannexins (Panx1 and Panx2), a family of integral membrane proteins, also express both rodent and human islets [67, 68]. Islet cell purification studies demonstrated that β-cell-rich fractions predominantly express the *Pnax2* mRNA, whereas the non-β-cell-rich fractions predominantly express the transcript of *Pnax1*, suggesting Pnax2 is selectively expressed in β-cells [67]; however, later on both *Panx1* and *Panx2* mRNA expression have been identified in both rodent and human islets [69]. While connexins form intercellular junctions between adjacent cells, pannexins form single hydrophilic channels that establish communication between the cytosol and the extracellular fluid, when opened [70]. These channels can be permeated by small molecules like glutamate, a putative β-to-α paracrine signal [71], such channels could operate in coordinating the antagonistic INS and GCG secretions.

### Adherent junctions

Islet endocrine cells also interconnected by cell adhesion molecules (CAMs) that are expressed on neighbouring cells and facilitate cell-to-cell adhesion. Most CAMs are glycoproteins having a molecular mass of about 120 KDa, functionally dependent on extracellular Ca^2+^ and thus are referred to as cadherins. These glycoproteins, which have a molecular mass of about 120 KDa, form a family including E, P, N, and R isoforms [72]. There are other CAMs, such as N-CAM also ensure cell–cell adhesion independently of Ca2 + [73]. Multiple CAMs are expressed in pancreatic islets to exert cell–cell communications. While N-CAM140 is preferentially found in non-β cell fraction, E-cadherin is the predominantly expressed CAM of β-cells [74]. Cadherin-mediated adhesion in single β-cells, but not α-cells, is positively regulated by glucose and it is associated with increased INS secretion [75].

### Tight junctions

β-cells are also connected with tetraspan transmembrane Claudin, which ise often associated with another membrane protein occludin and form tight junctions (TJs). TJs between neighbouring epithelial cells have important biochemical and physiological roles in islets as they allow selective and critical permeability to important compounds [76]. The transcriptome data sets and other approaches have demonstrated the involvement of previously understudied claudin (*Cldn*) family genes of TJs in the islets [77]. β-cells maintain a consistent orientation with respect to islet capillary networks, which is defined as ‘polarity of β-cells’ and the positioning of the polarity is determined by the expression of Par family protein Par-3 [17]. Par-3 is found at TJs in epithelial cells [78]; however, the 3D spatial distribution of β-cells within islets has revealed that Par-3 is specifically enriched in the apical region of β-cells positioned in a domain away from capillaries [17]. Thus, a critical function for Par-3 in the TJ assembly demonstrates the novel mechanism through which the spatial regulation between endocrine cells within islets is maintained.

### Focal adhesions

Interactions between endocrine cells and the extra cellular matrix are also critical for the normal development, survival, and function of β-cells. Integrins appear to be the primary receptors involved in cell–matrix interactions in islets, which influence islet vascular remodelling [79]. Integrins also facilitates the motility of endocrine cells within islets, which is necessary for the development of normal islet architecture and INS secretion [80]. Islets are richly endowed with capillaries and the majority of β-cells have at least one point of contact with the basement membrane (BM) of the capillary bed, occupying ~ 15% of the total β-cells surface area of β-cells [17, 81]. However, in contrast to the mouse islet, where endocrine cells are in direct contact with the vascular BM, the human islet has a unique double BM structure consisting of specifically structured and closely associated parenchymal and endothelial BMs [82]. Several non-integrin receptors such as the dystroglycan protein complex and Lutheran blood group glycoprotein (Lut), which are exclusively expressed on human islet cell membranes interact with BM of capillary networks and trigger the local responses of endocrine cells [82, 83].

### Functional heterogeneity of β-cells: Role of homotypic and the heterotypic interactions of cell adhesion molecules

Human β-cells exhibit functional heterogeneity in terms of their insulin secretory activity. This variability is influenced by intercellular communication among subpopulations of β-cells within the islet, both homologous and heterologous [84, 85]. Functional heterogeneity of β-cells also depends on the coordination of gap junctional communications demonstrated by a study where in isolated single cells, increasing glucose concentrations leads to increasing recruitment in the numbers of cells that responded to glucose [86, 87]. Thus, the activation of a single β-cell by glucose results in the recruitment of neighbouring β-cells to an active state where they can optimally utilize glucose [88]. This movement of heterogenous β-cells within the islets is mediated by cell adhesion molecules, CAMs, that significantly impact the functional heterogeneity of pancreatic β-cells.

Human pancreatic β-cells express CAMs, such as E-cadherin and N-cadherin, which mediate homotypic interactions between the cells. E-cadherin plays a critical role in regulating the adhesive properties of β-cells, which are essential for the aggregation of neighbouring endocrine cells into islets [89]. This creates a tight junction that seals the intercellular space and prevents the leakage of hormones. E-cadherin also participates in signaling pathways that regulate human β-cell survival, proliferation, and function [75, 90]. Another type of CAMs expressed by human β-cell is N-cadherin, which also mediates homotypic interactions between β-cells. N-cadherin predominantly expressed on the cell surface of human β-cells [90]. Under low glucose conditions, cadherins do not affect insulin secretion. However, at high glucose levels, both E- and N-cadherin increase insulin secretion from a single human β-cell, suggesting that they modulate the functional heterogeneity of human β-cells [75, 91]. The molecular mechanism of cadherins mediated enhancement of human β-cell function is still under investigation, but it is believed that changes in cortical actin distribution after cadherin engagement may play an important role. Furthermore, cadherin-mediated adhesion is increased by ROCK inhibitor, suggesting the involvement of Rho-GTPase pathway in cadherin-mediated modulation of human β-cell function [75].

Heterotypic interactions of CAMs in human pancreatic β-cells are complex and dynamic processes that can modulate the function and survival of β-cells by influencing their polarity, cytoskeleton, signaling pathways and gene expression. Integrins, a family of heterodimeric receptors that bind to various ligands on the extra cellular matrix (ECM) or on other cell surfaces [92] and mediates the heterodimeric interactions in β-cells. Integrins are expressed by all islet cell types, but their expression pattern and function vary among different β-cell subpopulations. For example, integrin, alpha 1 (ITGA1) has been reported to be significantly expressed in human β-cells, but with differential expression in a distinct subtype (ST8SIA1^±^ subtypes) of β-cell [37], suggesting the critical role of CAMs in the heterogeneity of human β-cells.

### Autocrine actions of pancreatic islet β-cells

The homeostasis of the islet endocrine system is maintained by a wide range of extracellular factors secreted from the same cells (autocrine action) or neighbouring cells (paracrine action). The signaling molecules that harmonize the physiological activities between the islet endocrine cells include hormones, peptides, neurotransmitters, proteins, and ions that act via specific receptors to elicit cellular responses. The autocrine signaling molecules in islet cells are often determined by the components of the synthetic machinery producing the molecule. For example, ATP, an important extracellular messenger molecule in the brain as well as in the vasculature and in endocrine organs, is also released during high glucose stimulation [93, 94]. Human β-cells express both ionotropic purinergic receptors P2X and metabotropic P2Y receptors; however, in response to glucose ATP is released from human β- cells and predominantly activates the P2X receptor to establish a positive feedback loop [95]. Thus, ATP signaling increases human β-cell electrical activity and promotes INS secretion [96, 97]. The nonessential amino acid glycine acts as an autocrine regulator of INS secretion in pancreatic β cells. Glycine receptors (GlyRs) are ligand-gated chloride ion channels that mediate fast inhibitory neurotransmission in the spinal cord and the brainstem [98]. Previous study demonstrated that GlyR is highly expressed in human β-cells and that activation of GlyR stimulates electrical activity. Upon stimulation and rise in cytosolic [Ca^2+^]i, intracellular glycine is released and serves as a positive autocrine signal for INS secretion [99].

Dopamine is a neurotransmitter that plays a fundamental role in many specific regions of the brain. β-cells synthesize dopamine from circulating l-dopa, store it in secretory vesicles, and release it with INS in response to glucose stimulation [100]. Dopamine type 2 receptors (D2R) are present in islets and dopaminergic feedback loop modulates their activation by dopamine to inhibit glucose-induced INS secretion from β-cells [101]. The monoamine neurotransmitter Serotonin (5-hydroxytryptamine, 5-HT) is produced in β-cells and released in response to glucose stimulation [102, 103]. In the absence of exogenous Serotonin, glucose-induced INS secretion is increased by an agonist of 5HT2 receptors and completely suppressed by blockage of the receptors, suggesting β-cells derived serotonin acts as an autocrine negative regulator of INS secretion [103]. Gamma amino butyric acid (GABA) is a major inhibitory neurotransmitter in the central nervous system and its levels in human islets are as high as in the brain [104]. While β-cell-derived GABA exerts an autocrine-positive effect on INS secretion via GABA-A receptors, GABA-B receptors augmented a negative autocrine action in pancreatic β-cells [105, 106] (Table 2).

**Table 2:** Autocrine and paracrine actions of molecules in pancreatic endocrine cells

	Autocrine mediators of pancreatic β-cells		Paracrine mediators of pancreatic β-cells		Autocrine or Paracrine mediators of other endocrine cells	
	Mediator	Target receptor	Mediator	Target receptor	Mediator	Target receptor
Hormones	Dopamine	D2R	Glucagon	GCGR	PP	NPY4R (in δ-cell), PPYR1 (in α-cell)
			SST-14	SSTR-5		
	Serotonin	5HT1R	GLP-1	GLP1R		
			Ghrelin	GHSR		
Non-hormone mediators	GABA	GABA-A, GABA-B	Ach	M3R	Glutamate	Metabotropic R, AMPA/Kainate (Both in α-cell)
			Glutamate	Metabotropic receptor		
	ATP	P2X/P2Y				
	Glycine	GlyR				

*D2R* dopamine receptor 2, *5HT1R* 5-hydroxy tryptamine receptor 1, *GABA* Gamma amino butyric acid, *GABA-A/B* Gamma amino butyric acid receptor A/B, *ATP* Adenosine tri-phosphate, *P2X/P2Y* purinergic receptor 2X/2Y, *GlyR* Glycine receptor, *GCGR* glucagon receptor, *SST-14* somatostatin-14, *SSTR* somatostatin receptor, *GLP1* glucagon like peptide-1, *GLP1R* GLP-1 receptor, *Ach* acetylcholine, *M3R* muscarinic receptor, *PP* pancreatic polypeptide, *NPY4R* neuropeptide Y receptor subtype 4, *PPYR1* pancreatic polypeptide receptor subtype 1, *AMPA* α-Amino-3-hydroxy-5-methyl-4-isoxazolepropionic acid

### Paracrine actions of pancreatic islet cells

In contrast to islet autocrine signaling, where a cell secretes a signaling molecule that binds to receptors on the same cell or other cells of the same cell type, paracrine signals influence other cells within the same islet by diffusing through the interstitial space or circulating in a local blood vessel [107]. The common feature of islet endocrine cells is the release of molecules that serve as paracrine signals (Fig. 1). GCG is a major hyperglycemic hormone in the organism that counters the decrease in plasma glucose levels. GCG secretion is thought to provide the first line of defence in glucose counter regulation [108]. In human islets, most of β-cells directly appose α-cells, δ-cells, or both and the proximity of β-cells with α-cells further enables paracrine interactions [20] (Fig. 1). GCG and glucagon-like peptide-1 (GLP-1) derive from a common precursor, proglucagon, but the primary source of GLP-1 is the L cells of gastrointestinal tract from which it is released in a meal dependent manner [109]. It is well established that human β-cells express GCG and GLP-1 receptors, GCGR and GLP1R, respectively [43, 110] (Fig. 1) (Table 2). Interestingly, two-fold higher expression of GLP1R than GCGR has been reported in human β-cells [9], suggesting a higher affinity of GLP1 for human β-cells. However, at the physiological concentration, GCG can activate GLP1R, whereas GLP-1 does not act on GCGR [111]. The notion that intra-islet GCG functions as a paracrine regulator of INS secretion have been demonstrated by increased INS secretion from β-cells overexpressing GCG receptors [112, 113]. Other studies used humanized mouse model and human islets also revealed the strong insulinotropic action of GCG [114, 115]. While 10 nM GCG effectively increases INS secretion in the presence of 6 mM glucose [116], GLP-1 is consistently, although variably, effective at 1–100 nM to enhance INS secretion from human islets [117, 118].

The parasympathetic neurotransmitter acetylcholine (ACh) is also released from α-cells in human islets and functions as a paracrine signal that primes the β-cells to respond optimally to subsequent increases in glucose concentration [119]. Despite not all other studies agreed on the presence of ACh vesicles (vACh) in α-cells (Fig. 1) [120], the mechanism of Ach-mediated increase in INS secretion involves activation of muscarinic M3 receptors in human β-cells [121, 122]. PPY, the 36-amino acid peptide, secreted from pancreatic γ-cells is also one of the paracrine modulators in pancreatic islets. The frequency of γ-cells is higher in the head of the pancreas [123], where the cells are found to accompany the outer mantle of rodent islets or lining the capillaries in human islets [124]. The paracrine function of PPY on α- and δ-cells is exerted through two distinct types of receptor families expressed on respective cell surfaces. On GCG producing α-cells, PPY binds to G-protein coupled receptor PPYR1 [125], while NPY receptor family NPY4 receptor (NPY4R) has been reported as the specific receptor for the PPY in SST producing δ-cells [126] and inhibits both GCG and SST secretion. Glutamate is another amino acid that functions as paracrine and autocrine regulator of INS and GCG secretion from pancreatic β- and α-cells, respectively. Glutamate is negatively charged major excitatory neurotransmitter in the central nervous system. In pancreatic islets, glutamate is released from α-cells when ambient glucose decreases [127] and once released, glutamate can act on different cell types through three different types of membrane receptors: AMPA/kainate receptors, metabotropic receptors, and NMDA receptors [128]. Pancreatic endocrine cells express those receptors and the paracrine function of glutamate on islet endocrine cells solely depends on cell-specific interaction of glutamate with those receptors. For example, Glutamate has been reported to activate α-cells via AMPA/kainate receptors [127, 129, 130]; inhibit α-cells via metabotropic receptors [131]. In β-cells, glutamate binds to the metabotropic receptors to increase INS secretion [132].

Islet δ-cell derived SST potently inhibits INS and GCG release from pancreatic islets and its effects are mediated via five SST receptor subtypes (SSTR1–SSTR5) [133]. There are two major physiological ligands for SSTRs: SST-14 and SST-28, consisting of 14 and 28 amino acids, respectively, and both forms of SST play an important role in the regulation of INS and GCG release from the endocrine pancreas. While SST28 is secreted by the gut, SST14 is secreted by δ-cells in the stomach and islets in response to glucose and amino acids [134, 135]. By using quantitative double-label confocal fluorescence immunocytochemistry, it has been demonstrated that all five human SST receptor subtypes (hSSTR1-5) subtypes are variably expressed in islets, however, SSTR5 being colocalized in 87% of β-cells, whereas, SSTR2 is strongly colocalized with GCG in 89% of α-cells [136]. Consistent with those findings, studies in SSTRKO mice also revealed that SST-mediated inhibition of GCG release in islets is primarily mediated via SSTR2, whereas INS secretion is regulated primarily via SSTR5 [137].

Growth hormone secretory hormone, GHRL, an acylated 28-amino acid peptide is expressed in pancreatic islet cells as well as the stomach. GHRL mRNAs are expressed in the pancreas and islets in rats and humans [138]. To become biologically active, GHRL needs to be acylated and acylated-GHRL is also detected in rat islets using radioimmunoassay (RIA) [139]. Ghrelin receptor (GHSR) is expressed in rat and human β-cells [140, 141] and GHRL inhibits glucose-stimulated INS secretion (GSIS) from β-cell lines [142, 143]. Taken together, it has been suggested that GHRL is a paracrine modulator of INS secretion and exert INS-static action on pancreatic β-cells via the inhibitory signaling of GHSR.

### Autocrine and paracrine action of β-cells and functional heterogeneity

Endocrine cells within the pancreatic islets secrete a wide range of diffusible chemical messengers, the autocrine or paracrine factors, which via binding to cognate receptors, are able to evoke biological effects in the neighbouring cells. Experimental studies have shown that by synchronizing their secretory activity in response to electrical coupling, autocrine or paracrine signaling, islet β-cells exploit several pathways of cell-to-cell communication [144]. However, human β-cells show complex and heterogeneous electrophysiological responses to many factors including ion channel antagonists [145]. This is probably due to the variability in the number of specific channels between β-cells that has been modelled to generate variable oscillation patterns and responses thus affecting the regularity of membrane potential bursting and [Ca^2+^] oscillations [146–148]. One of the paracrine mediators of human β-cell is mediated by glucagon (released from α-cell) signaling, which activates human β-cell G protein-coupled receptors (GPCR), including glucagon receptor (GCGR), and glucagon-like peptide 1 receptor (GLP-1R) [114, 116]. Both glucagon and GLP-1 increase intracellular cAMP levels and elicit synchronous intracellular Ca2 + oscillation respectively in human β-cell [116, 149]. By utilizing optogenetics, based on the signature of Ca^2+^ dynamics, discrete functional subpopulations of β-cells have been identified [14]. Therefore, it is likely that these subpopulations of β-cells will show functional heterogeneity in controlling coordinated electrical regulation and electrical dynamics in response to paracrine actions of glucagon or GLP-1.

### Hub cell dynamics

**Fig. 2:**
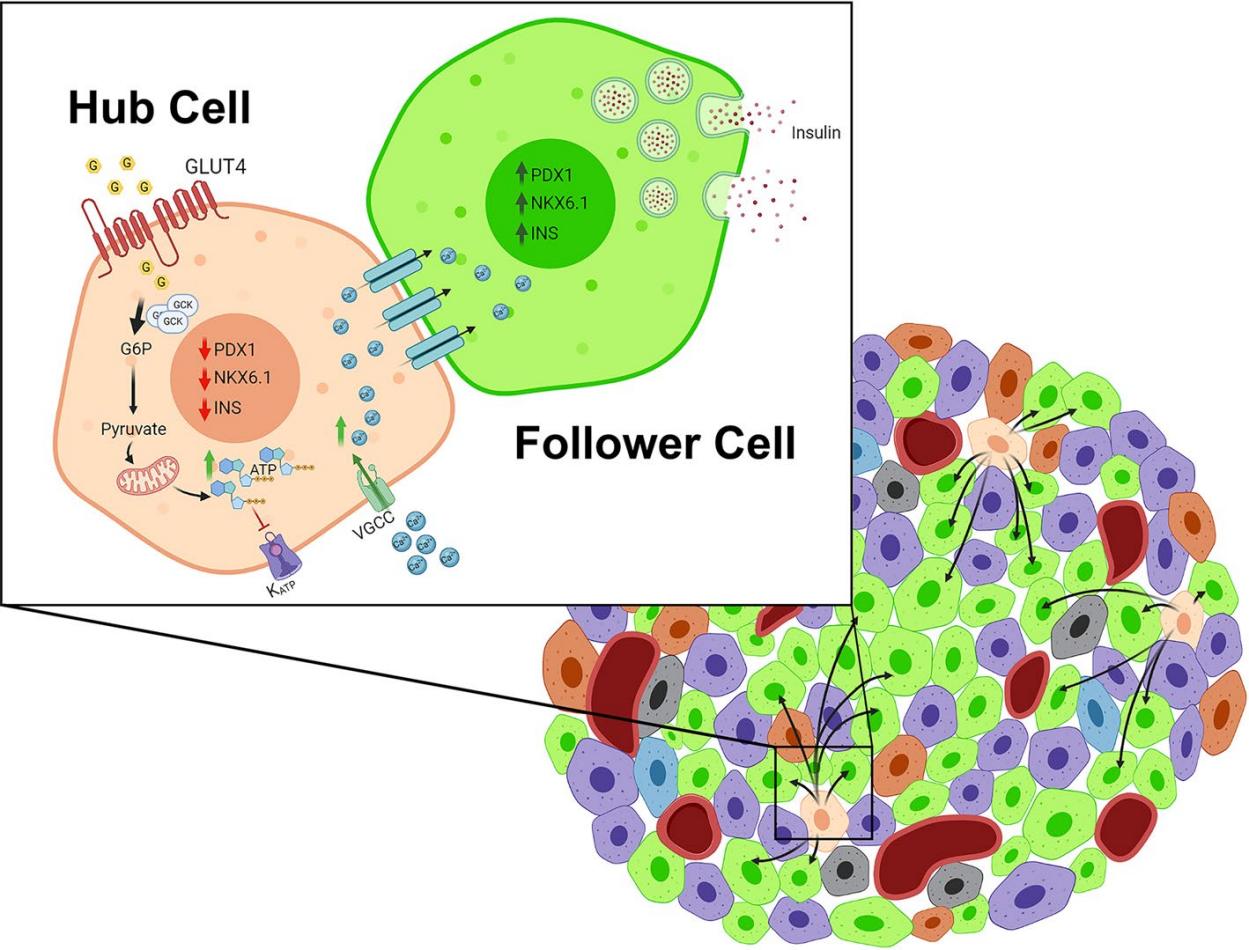
Schematic representation of pancreatic β-cell hub and follower cells dynamics. Hub cells characterized by the high expression of GCK, possibly due to lower PDX1 and NKX6.1 expression levels compared to follower cells. Hence, hub cells can respond faster to increased glucose levels by metabolizing glucose into pyruvate resulting in more ATP synthesis. The increase in ATP levels causes the closure of ATP-dependent potassium pumps, hence lowering intracellular positive charges, which consequently results in the opening of voltage-gated calcium channels (VGCC) and increases Ca^2+^ uptake by the cells. Later, the taken Ca^2+^ by hub cells can be transferred to follower cells through gap junctions causing stimulation of these cells, with high INS content, to release their stored INS. Hence, hub cells sense the changes in glucose levels more rapidly and take action to serve as ‘pace-maker’ cells

The idea of having pancreatic β-cells residing freely without clustering has been recently studied. Several studies have suggested that the role of these scattered β-cells (hub cells) is to regulate INS expression while coordinating islet oscillatory in a pacemaker-like manner (Fig. 2). A previous study employed large-scale functional cell mapping and optogenetics and demonstrated that ~ 1 to 10% of the β-cell population (i.e. hub cells) controls the synchronized and rhythmic action of pancreatic islets [150]. Hub cells have been found to be highly connected to other β-cells and robustly respond to high glucose levels compared to other “follower” β-cell subpopulations (Fig. 2). Hub cells have low expression of PDX1 and NKX6.1 pancreatic markers with increased expression of glucokinase (GCK), an important protein in the glycolysis pathway, but with less INS expression (~ 50%) [150, 151]. In addition, examination of the mitochondrial activity in these hub cells showed no difference in mitochondrial number, but increased activity is observed using tetramethylrhodamine ethyl ester (TMRE) imaging. The increased expression of GCK and mitochondrial activity in hub cells may suggest a mechanism of action for these cells for their high level of respondence to glucose levels. Furthermore, β-cells with low INS levels have been found to have high ATP levels with normal glucose sensing and cell survival [151]. This may suggest that hub cells conserve their energy by decreasing the ATP-consuming INS production process [151]. However, hub cells subjected to glucotoxicity, lipotoxicity or pro-inflammatory conditions, as in diabetes, fail to properly respond to the high glucose levels marking them as metabolically fragile cells, which eventually can lead to ER stress and cell dysfunction [150].

Interestingly, other subpopulations of β-cells exhibiting high functional connectivity, referred to as “wave-initiators” or “leaders” as stated in Sterk et al. [152] and “first responders” as explained in Kravet et al. [153], have been recently identified. It has been suggested that hub, wave-initiator, and first responder cells are characteristic components of islets; however, they exhibit distinct Ca^2+^ signaling characteristics and do not appear to have overlapping functions [152, 153]. The first responders lead the first phase Ca^2+^ response, are more excitable and crucial for mobilizing β-cells to increase Ca^2+^ immediately after glucose stimulation [153]. It has been found that during the transition from low to high glucose levels, the first responder cells play a crucial role in recruiting and coordinating Ca^2+^ activity within a specific time frame, as different cells lead distinct phases of the Ca^2+^ response to glucose (first responders for the first phase, and leader cells for the second phase) [153]. Previous studies have linked leader (wave origin) cells to the regulation of second-phase Ca^2+^ dynamics [152], in which hub cells are associated with maintaining elevated and coordinated Ca^2+^ [14, 154]. This suggests that the heterogeneity controlling second-phase Ca^2+^ is distinct from that of first-phase Ca^2+^.

There has been significant debate about the function of hub cells and other subpopulations and their effect on islet function. The identification and categorization of distinct β-cell subgroups, evaluation of their operational features, and understanding of how the group of cells work together to regulate intercellular calcium activity and insulin release within an islet are highly engaging areas of research that have captured significant interest within the islet biology field. Therefore, in the years to come, we anticipate an increase in research aimed at revealing the distinct contributions of these populations towards the coordinated β-cell activity under different conditions.

## Heterogeneity of stem cell-derived islet cells

### Generation of stem cell-derived islet organoids: recent progress

**Fig. 3:**
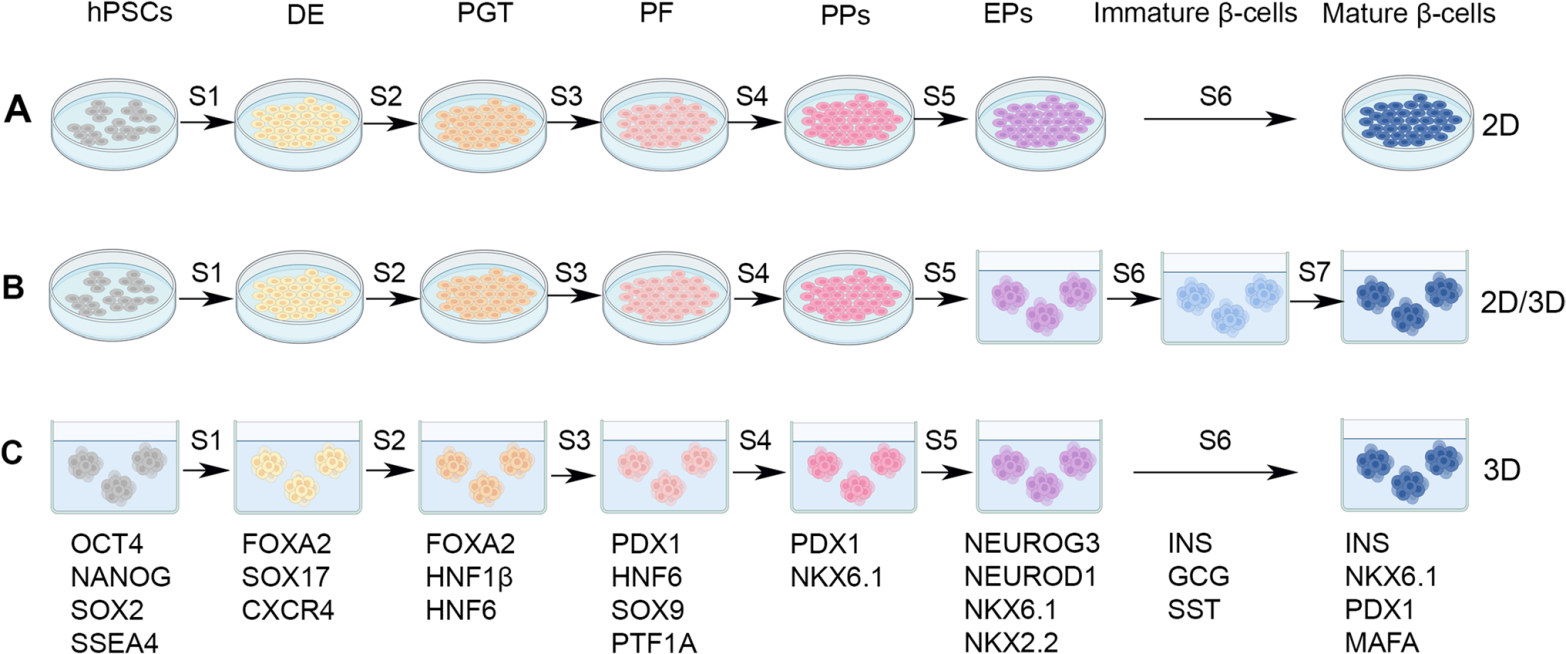
Overview of in vitro hPSC-derived pancreatic β-cell differentiation stages. hPSCs are differentiated into β-cells using a stepwise protocol of 7 stages each marked by the expression of specific transcriptional factors. **A** Some pancreatic differentiation protocols use the 2D culturing system from day 0 until the mature β-cell stage such as Hogrebe et al. [212]. **B** Other differentiation protocols use a 2D culturing system during the first 4 stages and then shift to 3D system during late stages (stages 5–7) to recapitulate the in vivo islet environment and allow better cell–cell contact such as Rezania et al. protocol [166]. **C** There are several protocols that start pancreatic differentiation as a 3D system from day 0 moving towards the last stages such as Melton’s protocols [166, 174]. *hPSCs* human pluripotent stem cells, *DE* definitive endoderm, *PGT* primitive gut tube, *PF*; posterior foregut, *PPs* pancreatic progenitors, *EPs* endocrine progenitors

Through utilizing hPSCs, different pancreatic differentiation protocols have been designed to mimic human embryonic development in vitro (Fig. 3). Understanding the sequential process of pancreatic development serves as the foundational template for protocols’ design. An initial step in pancreatic differentiation is to generate highly efficient definitive endoderm (DE) cells to be then directed towards hepatic or pancreatic tissues [155, 156]. Afterwards, activation of signaling pathways promoting pancreatic differentiation takes place while inhibiting hepatic-promoting signaling pathways [157]. To generate efficient DE, most protocols include Activin A in the first 3–5 days of differentiation to activate the Activin-Nodal signaling pathway [157, 158]. Generated DE cells are then introduced to FGF10, FGF7 or keratinocyte growth factor (KGF) and retinoic acid (RA) for FGF and RA signaling activation while inhibiting Sonic Hedgehog (SHH) signaling pathway through the addition of KAAD-Cyclopamine or SANT-1 for PDX1 induction [159–161]. An essential step then follows where hepatic lineage differentiation is prevented by inhibiting the BMP signaling pathway through BMP inhibitors such as Dorsomorphin or Noggin [162, 163]. Upon PDX1 induction, nicotinamide and EGF are added to induce NKX6.1 expression to generate PDX1^+^/NKX6.1^+^ pancreatic progenitors (PPs) [159, 160]. Following that, PPs are then differentiated into endocrine progenitors (EPs) expressing NEUROG3 through the addition of γ-secretase inhibitors or DAPT to inhibit the Notch signaling pathway, and β-cellulin and ALK5i to block the TGF-β signaling pathway [164]. To induce INS and MAFA production, high glucose media supplemented with Triiodothyronine (T_3_), a thyroid hormone, as well as ALK5i is added to the generated EPs to prevent their transition from epithelial to mesenchymal cells [165, 166]. In addition, NKX6.1 expression is found to be exclusively restricted to β-cells where it participates in their development, INS-containing vesicle formation, INS secretory function, and maintains their β-cell identity [167, 168]. As a result, the efficient generation of PDX1 and NKX6.1 co-expressing PPs and later generation of NKX6.1^+^/INS^+^ β-cells are crucial for the generation of mature, functional, mono-hormonal β-cells. Hence, the process of in vitro pancreatic β-cell differentiation utilizes small molecules and growth factors targeting specific pathways to either suppress or enhance them [4].

Multiple studies have shown that the generation of functional mono-hormonal pancreatic β-cells is mainly derived from PDX1 and NKX6.1 co-expressing PPs, which currently, can be generated with high efficiency (~ 80–90% of PDX1^+^/NKX6.1^+^ cells) [159, 169]. Constant modifications and protocol enhancements in pancreatic differentiation have led us to the generation of efficient functional pancreatic β-cells ~ 30–50% co-expressing C-PEP and NKX6.1 [165, 166, 170–174]. Yet, non-functional INS^+^ cells were also reported several times in which they were found to co-express GCG and/or SST; hence named, polyhormonal cells [175, 176]. In addition, these polyhormonal cells do not express several key pancreatic β-cell markers including, NKX6.1, MAFA, and important glucose channels/transporters that help in GSIS [165, 166, 172]. Despite protocol modifications, still, there are variations in hPSC-derived pancreatic β-cell differentiation from various cell lines due to the inherent hPSC line differences and efficiency of differentiation protocols [4]. Early pancreatic differentiation protocols depended on continuing β-cell maturation in vivo upon transplantation to animal models [165, 166]. However, recent protocols have provided improved differentiation protocols that are able to generate mature, functional β-cells in vitro [170, 171]. Recently, it has been found that the hippo-YAP signaling pathway affects β-cell differentiation [177]. Moreover, the inhibition of this specific pathway through the addition of Verteporfin, a YAP inhibitor, increases the number of NEUROG3 expressing EP cells and NKX6.1^+^/C-PEP^+^ β-cells; hence, enhancing β-cell differentiation and functionality [177]. In addition, the generation of functional β-cells has been found to be improved by emitting the use of growth factors and serum in stage 6 media and it would result in an increase in glucose responsiveness [174]. It is noteworthy that loss of ZnT8 has been observed to promote the maturation of hESC-dervied β-cells and protects them against cell death resulting from lipotoxicity or glucotoxicity. This is achieved by regulating zin levels, which reduces ER stress [178]. Although glucose responsive β-cells can now be generated, they still differ from adult human pancreatic β-cells in the transcriptomic profiles in which they show lower GLIS3, PCSK2, PAX6, and KCNK3 levels, in addition to lower calcium response [166]. Other studies tried to implement different culturing conditions, from 2D culturing system to 3D and found differences in the generated β-cell efficiency. A study identified that the re-clustering of hPSC-derived immature INS^+^ cells generate β-cells that resemble adult human β-cells, yet not entirely, as they express lower levels of maturity-associated markers UCN3, MAFA, and G6PC2 [165, 172]. Although current pancreatic differentiation protocols focus on eliminating the generated polyhormonal subset during in vitro differentiation, a subset of polyhormonal cells were found in human islets and most commonly in fetal islets [179]. In addition, multiple studies have reported the generation of large number of polyhormonal β-cells that resemble the transient endocrine cells observed during the mid-gestation of human fetal pancreas [162, 176, 180]. Yet, these polyhormonal cells lack the hallmark function of *bona fide* pancreatic β-cells, and do not secrete INS properly in response to glucose stimulation in vitro [5, 176]. In response to GSIS, in vitro generated polyhormonal β-cells resulted in varying degrees of glucose stimulation ranging from zero stimulation to 2-folds increase in INS secretion [176, 180, 181]. The generation of various numbers of polyhormonal cells in culture may be the cause of these variations and the reduced levels of insulin release. The in vitro generated polyhormonal cells may reflect the immature β-cells which are found during the mid-gestation period of human fetal pancreas [175]. Although the function of polyhormonal cells during human fetal development is not fully understood, immunohistochemical characterization suggested that these cells resemble the transcriptomic profile of α-cells [182]. Interestingly, slender T2D patients were found to have higher numbers of poly hormonal cells [183] that could play a role in stress-adaptation and maintaining normal glucose levels. Characterization of these poly hormonal cells revealed that these cells secrete INS and GCG upon non-glucose associated membrane depolarization and may be fated towards α-cells in vivo [182, 184–186]. The natural occurrence of these poly hormonal cells suggests that they might have a role in postnatal pancreatic β-cell maturation and function or other important roles that are yet to be discovered.

### Pancreatic progenitor and endocrine progenitor subpopulations derived from hPSCs

During embryo development, multipotent PP cells expressing the TFs, PDX1, NKX6.1, FOXA2, SOX9, HNF6, and PTF1A, differentiate into exocrine (acinar and duct) and endocrine (islets of Langerhans) pancreas [168]. PDX1 and NKX6.1 specifically, are known to be crucial factors for generating functional, mono-hormonal INS-secreting β-cells [2]. Therefore, hPSC differentiation protocols aim to generate efficient PP cells co-expressing PDX1 and NKX6.1. However, variations in PDX1 and NKX6.1 expression patterns have been observed when applying the same differentiation protocol on eight different human embryonic stem cell (hESC)/ human induced pluripotent stem cell (hiPSC) lines [2, 159]. This can be considered one of the factors resulting in mature β-cells heterogeneity that must be considered when designing transplantation protocols.

PDX1 is the earliest TF to be produced during pancreas development [187]. Interestingly, as PPs further differentiate, PDX1 expression becomes restricted to β-cells. Although PDX1 is essential for β-cell differentiation, generated PDX1^+^/NKX6.1^−^ PPs differentiate into GCG-secreting cells or non-functional poly hormonal β-cells that co-express INS and other islet hormones. These polyhormonal β-cells cannot be used as an option for transplantation therapy as they lack the hallmark function of β-cells [2, 5]. Multiple studies have reported the generation of GCG-secreting α-cells upon in vivo transplantation of hESC-derived poly hormonal cells [176, 184, 185, 188, 189]. Interestingly, a novel PDX1^−^/NKX6.1^+^ PP population has been recently identified and is able to generate functional glucose-responsive β-cells [168, 190].

Several in vitro pancreatic differentiation protocols suggest the generation of functional β-cells from PDX1^+^/NKX6.1^+^ PP populations that later express NEUROG3 [185, 186]. Studies have shownheterogeneity at EP stage marked by the difference in NEUROG3 expression where NEUROG3^–^ EPs lacked PDX1 expression but co-expressed CDX2 [191]. Another study further identified subclusters within NEUROG3^+^ cells: EPs (39.1%), poly hormonal endocrine cells (Endo; 42%), duct cells (6%), liver cells (8.2%), and an unknown cell type cluster (4.7%) [192]. The identified EP cluster had three different sub-clusters where they expressed different profiles with EP1 expressing the highest NEUROG3 levels, EP2 expressing TPH1 and FEV, and EP3 expressing GAST. In addition, the authors identified three endocrine clusters targeted towards different cell fates. Endo1 expressed ERO1B and SLC30A8 indicating β-cell fate, Endo2 expressed GCG, PEMT, and IRX2 indicating α-cell fate, while Endo3 expressed SST and HHEX suggesting δ-cell fate [192]. These results show the further complexity in endocrine cell populations and further heterogeneity can be observed as cells differentiate towards end-stages of pancreatic differentiation.

As hPSC-derived pancreatic differentiation protocols can generate heterogenous cell populations in which some could even be committed towards non-pancreatic lineages, several studies aimed to purify PPs directed towards β-cells. Previous studies reported that PPs can be purified using cell surface markers. Glycoprotein 2 (GP2), CD142, and CD24 have been found to be specifically expressed in PPs that can generate functional INS-secreting cells [186, 193–196]; therefore, those markers have been used to purify PDX1^+^ cells at the PP stage. On the other hand, NEUROG3-expressing cells can be isolated from the heterogenous population at the EP stage using specific surface markers, including SUSD2, CD318, CD133, CD200, and CD49f [186, 197]. This indicates that pure NEUROG3^+^ cells can be obtained for further differentiation into pancreatic islet cells. In 2018, a study compared in vivo and in vitro EP cells (stage 5) post-sorting based on E-cadherin, CD142 and SUSD2 surface markers which were previously described to discriminate between progenitors fated towards β-cells from those fated towards α- or γ-cells [196, 197]. In vitro EPs have increased levels of RFX6 and CDX2 that might reflect a mixed pancreas-duodenum fated populations or early PPs. On the other hand, human fetal EPs express high NKX6.1 and DLK1 levels. In addition, GPM6A, CDKN1A, and BMP5 have been found to be sporadically expressed in the in vitro-generated EPs [198].

### Subpopulations of hPSC-derived islet cells

Recently, multiple single-cell transcriptome studies performed on human islet cells and hPSC-derived islets highlighted the unique genetic signatures and intra-islet heterogeneity levels. By tackling these studies, it is suggested that the early stages of pancreatic development are widely consistent with homogenous profiles. On the other hand, during late pancreatic developmental stages where endocrine commitment takes place, this uniformity starts to be lost resulting in different end-stage cells with different genetic profiles. Although limited studies focused on generated pancreatic β-cell populations derived from hPSCs have been found, multiple single-cell analysis studies have identified and supported that islet cells heterogeneity starts from pancreatic and EP stages. These studies also indicated that hPSC-derived islets generated in vitro, differ from adult human islets where cell heterogeneity depends on the differentiation protocol used.

One of the important studies that tackled hPSC-derived β-cell heterogeneity has been done by Veres et al. [174]. The study reported that cells’ heterogeneity starts to appear from stage 4 and continued onwards. At end-stages, β-cells expressing β-cell markers such as INS, NKX6.1, and ISL1, α-like cells expressing GCG, ARX, IRX2, including INS, and a small subset of SST^+^/HHEX^+^/ISL1^+^ resembling δ-cell population have been identified [174]. They also detected a new population of enterochromaffin cells, located within the intestinal epithelium, marked by the expression of TPH1 and SLC18A1, which are normally not present in fetal or adult islets [174]. Several study groups have identified such off-target populations during hPSC-derived islet cell differentiation [174, 192, 199, 200]. Therefore, β-cell purification methods using surface markers can be used to isolate the functional mono-hormonal β-cell fraction from the total heterogenous hPSC-derived islet populations. Recent studies have identified CD49a as a specific cell surface marker for the functional INS^+^ cell fraction, which then can be re-aggregated to form 3D islet organoid structures [174, 201]. Nevertheless, the isolation of β-cells from the generated islet organoids would cause disturbance of the islet architecture that is required to mimic the in vivo environment. Reports have shown that disturbed islets perform less when compared with intact islet cells during GSIS [202–204]. Yet, islet cell fractionation and then re-aggregation of isolated β-cells enhances their functional maturation and response to glucose [172]. These results support the importance of the complex and heterogenous islet architecture and close proximity of cells in conferring β-cell functionality and response to glucose stimulation through β-cells contact with the environment and other types of endocrine cells. Other combinations of surface markers have been reported to enrich β-cell fraction from the whole pancreas including Hpi2^+^/HPα1^−^/HPx2^−^ and CD9^+^/CD56^+^, yet, a small fraction of δ-cells was also purified using these markers indicating the essential need for full characterization of the identified surface markers [205, 206].

### Heterogenous versus homogenous endocrine cell population for cell therapy

The organization of endocrine cells within the islet is crucial for the functional performance of pancreatic β-cells and response to glucose alterations. Studies have shown that intact islet cells respond more efficiently than isolated β-cells [202–204]. Within the human islet, the pancreatic β-cells are directly communicating with other endocrine cells for paracrine signaling and electrical coupling [19, 65]. This raises an important question regarding the use of purified β-cells versus a heterogenous islet cell population for transplantation therapy.

The secretion of GCG and GCG-derived peptides has been shown to enhance INS exocytosis machinery [207] and that ACh secreted from α-cells affects β-cell functionality [119]. In addition, SST secretion is known to inhibit INS secretion [208]. While other endocrine cells can affect β-cells functionality, β-cells can also have a direct effect on other endocrine cells. INS receptors are highly expressed in α-cells where excessive INS secretion can inhibit GCG secretion [209]. This interwind hormonal effect on other endocrine cells indicates a complex yet homogenous intra-islet activity where the different types of endocrine cells have effects on other endocrine cells’ functionalities. In cases of T2D, studies have shown increased α- to β-cell ratio as α-cells are more resistant to metabolic stresses through their high expression of anti-apoptotic proteins [210]. However, in T1D, a decrease in α-cell population is observed [211], which can correlate to an inter-cell dependency between α- and β-cells for cells’ development and function. Therefore, it is important to study co-transplanting β-, α-, and δ-cells together, which may give better outcomes in correcting hyperglycaemia. For cell transplantation therapy, researchers may need to design a cell product, which includes the main endocrine islet cells in addition to β-cells that can be achieve selecting for each endocrine cell type through specific surface markers and re-aggregating the purified islet cells.

## Conclusion and future perspectives

Pancreatic β-cell heterogeneity has recently been the focus of new pancreatic β-cell research. Recent advances in single-cell studies have paved the way for further understanding of human islet structure and cell composition. Heterogeneity of pancreatic β-cells can be observed in normal human islets, disease status, and more variability is seen in in vitro generated β-cells. The observed variability in pancreatic β-cells generated in vitro could be the cause for the slowly improved pancreatic differentiation protocols that are able to generate functional, mono-hormonal β-cells. Here, we tackled β-cell heterogeneity from different aspects while looking at their maturity status, under stress and diabetes conditions, as well as their abilities to respond to increased glucose levels. We further analysed inter- and intra-islet cell communication and its importance in developing a harmonious network of cell–cell communication. Furthermore, applying single analysis tools on the heterogenous populations will allow researchers to better understand the transcriptome profiles of different islet cell populations. By implementing newly developed research techniques such as single-cell sequencing, a better understanding of the different β-cell populations is within reach and more approachable.

## Data Availability

No data availability.

## Abbreviations

CAMs: Cell adhesion molecules
Cxs: Connexins
DE: Definitive endoderm
EPs: Endocrine progenitors
ER: Endoplasmic reticulum
GCG: Glucagon
GCK: Glucokinase
GJs: Gap junctions
GlyRs: Glycine receptors
hiPSCs: Human induced pluripotent stem cells
hPSCs: Human pluripotent stem cells
INS: Insulin
PPs: Pancreatic progenitors
ROS: Reactive oxygen species
SST: Somatostatin
T1D: Type 1 diabetes
T2D: Type 2 diabetes
TFs: Transcription factors
UPR: Unfolded protein response
